# Revolutionizing adjuvant development: harnessing AI for next-generation cancer vaccines

**DOI:** 10.3389/fimmu.2024.1438030

**Published:** 2024-08-14

**Authors:** Wan-Ying Zhang, Xiao-Li Zheng, Paolo Saul Coghi, Jun-Hui Chen, Bing-Jun Dong, Xing-Xing Fan

**Affiliations:** ^1^ Dr. Neher’s Biophysics Laboratory for Innovative Drug Discovery, State Key Laboratory of Quality Research in Chinese Medicine, Macau Institute for Applied Research in Medicine and Health, Macau University of Science and Technology, Macao, Macao SAR, China; ^2^ Intervention and Cell Therapy Center, Peking University Shenzhen Hospital, Shenzhen, China; ^3^ Gynecology Department, Zhuhai Hospital of Integrated Traditional Chinese and Western Medicine, Zhuhai, China

**Keywords:** AI drug discovery, vaccine adjuvant, cancer, immune agonist, small molecular

## Abstract

With the COVID-19 pandemic, the importance of vaccines has been widely recognized and has led to increased research and development efforts. Vaccines also play a crucial role in cancer treatment by activating the immune system to target and destroy cancer cells. However, enhancing the efficacy of cancer vaccines remains a challenge. Adjuvants, which enhance the immune response to antigens and improve vaccine effectiveness, have faced limitations in recent years, resulting in few novel adjuvants being identified. The advancement of artificial intelligence (AI) technology in drug development has provided a foundation for adjuvant screening and application, leading to a diversification of adjuvants. This article reviews the significant role of tumor vaccines in basic research and clinical treatment and explores the use of AI technology to screen novel adjuvants from databases. The findings of this review offer valuable insights for the development of new adjuvants for next-generation vaccines.

## 1 Introduction

In the field of drug discovery and screening, artificial intelligence (AI) has become a dominant method in the community due to its unique advantages (1). Firstly, the AI-based process significantly reduces the time and resources required for the research because it can quickly screen, design, and optimize millions of compound structures with enhanced prediction accuracy (2, 3). Moreover, AI facilitates studying specific targets and discovering suitable target compounds, often called the “automation” process (4). Cancer vaccines that use tumor antigens and adjuvants to identify and eliminate cancer cells are becoming a promising approach (5, 6). As the main component of cancer vaccines, adjuvants mainly enhance the vaccine’s immune response speed, intensity, and persistence (7). At present, a variety of proteins/receptors are being studied as potential targets for vaccine adjuvant design, including Toll-like receptor (TLR) (8, 9), Stimulator of interferon genes (STING) (10, 11), Indoleamine 2, 3-dioxygenase (IDO) (12), Programmed Cell Death Protein 1 (PD-1)/Programmed Cell Death Ligand 1 (PD-L1) (13) and so on. Although many potential adjuvants have been evaluated in preclinical studies, the number of adjuvants successfully converted to approved vaccines is still minimal (14). With the researchers’ mastery of the immune system and vaccine technology, it has been found that the classical licensed adjuvants have limited applicability to cancer antigens, especially nucleic acid antigens, because of their inefficiency. It can no longer meet the requirements of current cancer immunotherapy. Therefore, the integration of AI technology and tools to promote the generation of novel adjuvants is required. The limitations of adjuvants have many causes, including the small number of currently approved adjuvants for clinical use and the significant barriers to translating new adjuvants from the laboratory to clinical applications. The high cost of clinical approval for new adjuvants and the preference of developers for existing adjuvants due to their lower regulatory risk are also contributing factors (15). To address this issue, the Adjuvant Development Program of the National Institutes of Health (NIH) has provided crucial funding and support for the development and application of new adjuvants in recent years (16, 17). Another pressing issue is the low response rate of adjuvants. Specifically, why do adjuvants added to vaccines fail to work effectively or meet expectations? In our literature review, we found that this problem is mainly because many adjuvants do not translate well from animal studies to clinical studies. For example, there are significant differences in TLRs (Toll-like receptors) between different species (18). Therefore, it is crucial to screen potential TLR agonists in appropriate animal models while considering species differences. AI and machine learning are transforming multiple fields, including vaccine adjuvant development. For instance, in the study of new human TLR9 ligands, A. Ahuja et al. developed a customized software program called the ‘Synthetic Chemist.’ Specifically, 10^16^ synthetic oligonucleotides were generated using a computer, and a second machine learning program, ‘Search Algorithms for Ligands,’ was used to screen these ligands to find the best pan-species TLR9 ligand (CPG55.2) (19, 20). Subsequently, CPG55.2 was shown to be active across multiple species and successfully used in the development of vaccines for influenza (21), malaria (22), herpes simplex virus type 2 infection (23), poliovirus (24), and COVID-19 (25, 26). This example illustrates the advantages of using AI to screen adjuvants and demonstrates how the developed software has played an important role in advancing new adjuvants in practical applications. This article summarizes prospective tactics and technology for generating cancer vaccine adjuvants, which can be helpful in the development of cancer vaccines.

## 2 Status and challenges of cancer vaccine adjuvants

Cancer vaccine adjuvants are substances used in cancer vaccines to enhance the immune system’s response to antigens. They play a crucial role in improving the efficacy of cancer vaccines by enhancing the body’s immune response to cancer cells. Like an experienced coach, they are gradually rewriting the rules of immunotherapy (27). Adjuvants can help present antigens to the immune system, stimulate the production of cytokines, and activate various immune cells, making them an essential component in the development of effective cancer vaccines (28).In recent years, scientists have been continuously exploring how to best utilize these “coaches” to enhance patients’ immune responses through precise immunological data and personalized treatment plans. To ensure safety and efficacy, ideal cancer vaccine adjuvants should possess several key characteristics: low or no toxicity, minimal side effects, the ability to enhance the immune response to vaccine antigens, specificity, stability, and ease of large-scale production. In a groundbreaking study, researchers developed a nanoparticle-based adjuvant that showed remarkable results in clinical trials, achieving unprecedented therapeutic outcomes by enhancing antigen presentation and stimulating immune cells (29, 30). Cancer vaccine adjuvants can be broadly classified based on their mechanisms and sources into the following categories: biological adjuvants, chemical adjuvants, particulate adjuvants, and combination adjuvants.

### 2.1 Biological adjuvants

Biological adjuvants are natural or synthetic biological molecules that enhance vaccine efficacy by modulating the immune system. These adjuvants can enhance immune responses through various mechanisms, including enhancing antigen presentation, activating immune cells, and promoting cytokine production.

#### 2.1.1 Cytokines

GM-CSF (Granulocyte-Macrophage Colony-Stimulating Factor) is an important cytokine that enhances immune responses by promoting the maturation and function of dendritic cells and macrophages. It is widely used in cancer vaccines to enhance antigen presentation and T cell activation (31). Provenge (Sipuleucel-T), the first FDA-approved therapeutic cancer vaccine, uses GM-CSF as an adjuvant to enhance antigen presentation by dendritic cells, used for treating prostate cancer (32). DCVax^®^-L, a personalized dendritic cell vaccine for glioblastoma, combines the patient’s own tumor antigens with GM-CSF, significantly extending patient survival (33).

IL-12 (Interleukin-12) is another key cytokine that induces Th1 immune responses, promoting the activation of cytotoxic T cells and NK cells, thereby enhancing immune attacks on tumor cells. NKTR-214 (Bempegaldesleukin) is an IL-2 pathway agonist that enhances T cell and NK cell activity. It is used in combination with immune checkpoint inhibitors to treat various solid tumors and can provide long-lasting immune responses to prevent tumor recurrence when combined with anti-CTLA-4 antibodies (34).

#### 2.1.2 Microbial components

BCG (Bacillus Calmette-Guérin) is an attenuated live vaccine derived from Mycobacterium bovis, widely used for bladder cancer immunotherapy (35). Initially developed to prevent tuberculosis, BCG is used as an immunoadjuvant in cancer immunotherapy due to its ability to stimulate the innate immune system, activate macrophages and dendritic cells, and promote antigen presentation and specific T cell responses (36).

CpG (Cytosine-phosphate-Guanine) oligodeoxynucleotides are synthetic molecules that activate TLR9, inducing strong Th1 immune responses, promoting dendritic cell maturation, and enhancing antigen presentation. Several new adjuvants, including TLR3, TLR7, and TLR9 agonists, are undergoing clinical trials to enhance the efficacy of immune checkpoint inhibitors and improve cancer vaccine responses (37).

#### 2.1.3 Other biological molecules

Mitogens, such as superantigens and bacterial toxins, can strongly activate T cells and enhance immune responses. For example, Tetanus Toxoid Fragment C (TTFC) is used as an effective adjuvant to enhance the efficacy of various vaccines (38).

### 2.2 Chemical adjuvants

Chemical adjuvants are synthetically designed substances aimed at enhancing immune responses. They improve vaccine efficacy through various mechanisms, including enhancing antigen uptake, activating immune cells, and promoting inflammatory responses. The in-depth study and application of chemical adjuvants have significantly improved vaccine efficacy, providing more options and possibilities, especially in cancer vaccine development.

#### 2.2.1 Aluminum salts (Alum)

Aluminum salts are among the earliest and most widely used vaccine adjuvants. They enhance immune responses by forming an antigen depot, prolonging antigen presence in the body, and promoting uptake by antigen-presenting cells (APCs) (39). Aluminum salts are widely used in various vaccines, including DTP (Diphtheria, Tetanus, Pertussis) vaccines (40), hepatitis B vaccines (41), and human papillomavirus (HPV) vaccines (42). In some cancer vaccines, aluminum salts are used as adjuvants to enhance immune responses. For instance, aluminum adjuvants in HPV vaccines such as Gardasil and Cervarix improve vaccine immunogenicity and effectively prevent HPV-related cancers (43).

#### 2.2.2 Saponins

Saponins, such as Quillaja saponaria extract QS-21, are the only saponin adjuvant approved for clinical use in humans and have demonstrated strong immunostimulatory activity. Traditional extraction methods for QS-21 are time-consuming and yield low amounts (44). Recently, Liu et al. developed a novel method to synthesize QS-21 analogs, laying a rational foundation for effective vaccine adjuvants (45).

### 2.3 Particulate adjuvants

#### 2.3.1 PLGA nanoparticles

PLGA (Polylactic-co-glycolic acid)) is a biodegradable polymer commonly used to prepare nanoparticles. PLGA nanoparticles enhance immune responses by controlling antigen release rates, increasing antigen stability, and promoting uptake by antigen-presenting cells (46). PLGA nanoparticles have been studied in various vaccines, including rabies and tuberculosis vaccines, showing significant immune-enhancing effects (47, 48). Additionally, PLGA is often used as a stabilizer for solid particle-stabilized emulsions (Pickering emulsions) (49). Due to its high stability, biocompatibility, and large loading capacity, Pickering emulsions have been widely used in biomedicine. PLGA-based Pickering emulsions can adhere to cell membranes, increasing contact areas (50).

#### 2.3.2 Liposomes

Liposomes are tiny vesicles formed by phospholipid bilayers that can effectively encapsulate and deliver antigens and adjuvants. Liposomes enhance immune responses by improving antigen delivery, promoting uptake by antigen-presenting cells, and providing antigen protection (51). Liposomes are widely used in various vaccines, including influenza and COVID-19 vaccines. For example, mRNA vaccines (such as Pfizer-BioNTech’s COVID-19 vaccine) use liposomes as delivery systems, significantly improving vaccine immunogenicity and protective efficacy (52). Liposome adjuvants show promising applications in vaccine development. For instance, a study indicated that using liposomes as adjuvants in influenza vaccines significantly improved immune responses in elderly populations (53).

### 2.4 Combination adjuvants

Combination adjuvants use multiple adjuvants together to achieve synergistic effects and enhance vaccine immune responses. This strategy aims to utilize complementary mechanisms of different adjuvants to provide stronger and longer-lasting immune protection compared to single adjuvants.

#### 2.4.1 Combination of cytokines and nanoparticles

Cytokines such as GM-CSF can enhance the function of antigen-presenting cells (APCs) and promote antigen presentation. Nanoparticles can efficiently deliver antigens and adjuvants to immune cells and provide sustained immune stimulation through controlled release mechanisms. A study combined GM-CSF with PLGA nanoparticles for melanoma vaccine development, showing that this combination significantly enhanced vaccine immunogenicity and anti-tumor efficacy (54).

#### 2.4.2 Combination of multiple nanoparticles

Different types of nanoparticles (e.g., gold nanoparticles and PLGA nanoparticles) can enhance immune responses through different mechanisms. Gold nanoparticles can improve antigen stability and delivery efficiency, while PLGA nanoparticles provide sustained immune stimulation. Research indicated that combining gold nanoparticles and PLGA nanoparticles in liver cancer vaccines significantly enhanced antigen-specific T cell responses and anti-tumor activity (55).

#### 2.4.3 Combination of TLR agonists and liposomes

TLR agonists (e.g., CpG oligodeoxynucleotides) can activate the innate immune system, promote antigen presentation, and T cell activation. Liposomes, as delivery systems, can effectively encapsulate and deliver antigens and adjuvants, protecting them from degradation. In HPV-related cancer vaccine research, combining TLR9 agonist CpG with liposomes significantly enhanced vaccine immune responses and protective efficacy. MPL, a TLR4 ligand derived from LPS, enhances the ability to prevent tuberculosis in mice and crab-eating monkeys when present in the Ag85B-ESAT-6—DDA liposome formulation (56).

Recent research has explored various novel adjuvants and delivery systems to enhance the efficacy of cancer vaccines. For instance, studies have shown that using bispecific antibodies (i.e., antibodies that target both tumor cells and immune cells simultaneously) can enhance immune responses against cancer. Bispecific antibodies have demonstrated significant potential in targeting tumor-associated antigens and activating immune cells, thereby improving therapeutic outcomes (57).

Despite the crucial role of adjuvants in vaccine development, their use and development still face many challenges. Safety issues have always been paramount in drug development, as many adjuvants can cause local or systemic reactions or autoimmune diseases. Therefore, it is necessary to develop new adjuvants that reduce side effects and increase safety. For example, optimizing dosage and administration methods, as well as conducting extensive clinical trials for validation, are important. Individual differences in immune responses also need to be considered. Due to differences in genetics (58), environment (59), age (60), sex (61), occupation (62), and other factors, individuals may exhibit varying intensities and types of immune responses when receiving the same vaccine or being exposed to the same pathogen.

Additionally, the production processes for adjuvants are complex, and quality control often presents significant challenges, with variations between different batches. Thus, it is crucial to establish and adhere to strict standardized procedures and employ advanced quality control techniques. Besides these issues, adjuvants also face challenges such as insufficient immune persistence, high costs, and inadequate regulation, all of which need to be addressed and improved.

## 3 Online available algorithms and databases of AI Drug Discovery

Recently, many algorithms and databases have been rapidly established for AIDD. For example, DeepChem, a Multi-Layer Perceptron (MLP) model primarily using Python AI system to search for suitable drug candidates in drug discovery (63), and DeepTox, a software capable of predicting the toxicity of 12,000 drugs (64). For the development of small molecule drugs, there is a database (https://smpdb.ca/) that includes 49,833 pathways that can be found in databases that are not accessible through other routes (65). Furthermore, Shtar and colleagues have established the continuously updated Continuous Drug Combination Database (CDCDB). In this database, they use various methods, including natural language processing, to improve the drug combination discovery process and ensure the database is applicable for predicting drug synergies (66). In sum, we have summarized other related databases and open-source tools in **Table 1**.

**Table 1:** Databases and open-source tools.

Tools	Explanation	Github/website	Ref
GoPubMed	A specialized search engine for PubMed articles, using text mining to find relevant research papers	http://www.gopubmed.org	(67)
BioRAT	A tool for searching full-text articles to mine text data for useful information	http://bioinf.cs.ucl.ac.uk/biorat/	(68)
DeepChem	A machine learning tool in Python to help find potential drug candidates	https://github.com/deepchem/deepchem	(63)
DeepNeuralNetQSAR	Uses computational tools to predict how compounds will behave based on their molecular structure	https://github.com/Merck/DeepNeuralNet-QSAR	(69)
DeepTox	Software that predicts the toxicity of around 12,000 drugs	www.bioinf.jku.at/research/DeepTox	(64)
GeneWays	Extracts information about biological pathways from scientific texts.	http://geneways.genomeleft.columbia.edu	(70)
PotentialNet	Uses neural networks to predict how well ligands (small molecules) will bind to their targets	https://pubs.acs.org/doi/full/10.1021/acscentsci.8b00507	(71)
ORGANIC	Generates new molecules with desired properties	https://github.com/aspuru-guzik-group/ORGANIC	(72)
CancerDR	Database of cancer drug resistance, listing the effectiveness of 148 anticancer drugs on nearly 1,000 cancer cell lines	http://crdd.osdd.net/raghava/cancerdr/	(73)
PubChem	A database of chemical information and their biological activities	https://pubchem.ncbi.nlm.nih.gov	(74)
BRENDA	Comprehensive database of drug and drug target information	http://www.brenda-enzymes.org	(75, 76)
DrugBank	Comprehensive drug–target and drug data information database	http://www.drugbank.ca	(77)
ChEMBL	Database of bioactive molecules with drug-like properties, based on published research	https://www.ebi.ac.uk/chembl	(78)
ChEBI	Database of chemical entities of biological interest	http://www.ebi.ac.uk/chebi	(79)
ZINC	Database containing curated chemical compounds > 750 Million compounds	http://zinc.docking.org/	(80)
Chemputer	Give detailed recipe for compound synthesis	https://zenodo.org/record/1481731	(81)
Chemical VAE	AUses variational autoencoders to automatically design new chemicals	https://github.com/aspuru-guzik-group/chemical_vae	(82)
VAE	Improve the chemical property prediction performance of machine learning models	https://github.com/znavoyan/vae-embeddings	(83)
Co-VAE	A tool that predicts drug-target affinity better than existing methods	https://ieeexplore.ieee.org/document/9576631	(84)
Cloud 3D-QSAR	A web tool for building models that relate chemical structures to their biological activities	http://agroda.gzu.edu.cn:9999/ccb/server/cloud3dQSAR/	(85)
Neural graph fingerprint	Uses convolutional neural networks to predict properties of new compounds	https://github.com/HIPS/neural-fingerprint	(86)
BindingDB	Database of experimental data on how small molecules interact with proteins	http://www.bindingdb.org	(87)
Therapeutic target database	Comprehensive resource on therapeutic protein and nucleic acid targets, diseases, pathways, and drugs	https://db.idrblab.net/ttd/	(88)
Cambridge structural database (CSD)	Repository for small molecule organic and metal-organic crystal structures	https://www.ccdc.cam.ac.uk/solutions/software/csd/	(89, 90)
NCI open database	Database from the National Cancer Institute with a wide range of chemical compounds	https://cactus.nci.nih.gov/download/nci/	(91)
ENAMINE database	The world’s largest collection of novel building blocks (more than 210 million) 36B Billion REAL compounds and Custom Library Synthesis	https://enamine.net/	(92)
CHEMBRIDGE Database	Database of over 1.3 million small molecules for screening, including unique macrocycles and building blocks	https://chembridge.com/	N/A
AMTDB	Open-access repository specifically for anti-tumor autophagy modulators	https://amtdb.vercel.app/	(93)
ChemSpider	Chemical database maintained by the Royal Society of Chemistry with extensive chemical information.	http://www.chemspider.com/	(94)
GRAC	Database focused on compounds related to G Protein-Coupled Receptors (GPCRs)	http://www.guidetopharmacology.org/about.jsp	N/A

N/A, Not Available.

## 4 AIDD assists in the discovery of novel adjuvants

Depending on different technical routes and functions, AIDD could be divided into five subgroups, including *De novo* design, Virtual screening, Properties and Toxicity prediction, and Drug repurposing.

### 4.1 The “*De novo* design” in the discovery of adjuvant

The stages of drug design have always been a focal point in AI. “*De novo* design” is an indispensable aspect of using AI in drug development. Since the introduction of the first “*De novo* design” software, HSITE/2D Skeletons, which was developed in the 1980s (95–97), it has gradually replaced traditional methods in drug discovery design, encompassing scoring, assembly, and search strategies. It has been continuously used until today (98). Based on it, Popova et al. designed a reinforcement deep learning algorithm called ReLeaSE (https://github.com/isayev/ReLeaSE). This algorithm includes deep neural networks (DNN) for compound generation and prediction of compound properties (99). Open-sourced tools for finding the optimal pathways of target molecule synthesis were also available, called AiZynthFinder (https://github.com/MolecularAI/aizynthfinder) (100).

To focus on exploring small molecule adjuvants for cancer vaccines, a practical and highly efficient tool to find specific inhibitors or agonists is necessary. Zhavoronkov et al. designed a tool called Generative Tensor Reinforcement Learning (GENTRL) for small molecule “*De novo* design”. GENTRL optimizes synthesis feasibility, novelty, and biological activity. Using GENTRL, they discovered a potent inhibitor of Discoidin Domain Receptor 1 (DDR1), a kinase target associated with fibrosis and other diseases, within 21 days (101). Chemistry42 is a platform for *de novo* small molecule design and optimization. It combines AI and medicinal chemistry to generate novel structural molecules efficiently with *in vitro* and *in vivo* validation. These approaches have effectively validated for DDR1 and CDK20 (102). All these advancements provide significant assistance to AIDD or adjuvant identification.

### 4.2 Virtual screening

Virtual screening technology is widely applied to facilitate the screening of large compound libraries based on computer simulations and molecular modeling techniques (103), thus contributing to drug high-throughput screening (HTS) (104). It allows for assessing the biological activity of candidate compounds based on their chemical structures, thus predicting their interactions with specific targets (105). Therefore, it enables the rapid identification of potentially active compounds, saving time and resources during the early stages of drug development (106). Traditional HTS often faces high costs, time-consuming processes, and limited sample quantities (107). This fast and efficient screening provides crucial support to drug development and advances the progress of drug discovery (108).

There are two main technical routes for virtual screening, including Ligand-based or Structure-based virtual screening (LB/SBVS), which are all primarily attributed to the advancements in extensive databases of protein and chemical structures. With improved computational power and the accumulation of large compound libraries, virtual screening can more accurately predict the biological activity and efficacy of candidate compounds (109). This has led to successful discoveries of new bioactive molecules, particularly in drug repurposing (110).

#### 4.2.1 Ligand-based virtual screening

LBVS is a computer-assisted drug discovery method that predicts compounds’ binding ability and affinity by simulating their interactions with protein targets (111). This screening approach is commonly used to identify potential drug candidates, particularly for drug discovery targeting known protein targets. It typically involves data preparation, ligand preprocessing, target preprocessing, molecular docking, and result analysis (112). For instance, Luca et al. recently utilized LBVS to identify an effective α-Syn amyloid formation inhibitor, MeSC-04 and demonstrated its binding mode. This work provided new insights for developing α-Syn amyloid inhibitors from synthetic sources (113). Zarei et al. employed LBVS on the RON receptor homology model to screen the ZINC database, identifying two compounds, TKI1 and TKI2, further evaluated *in vitro*. This study laid the foundation for novel RON inhibitors applicable in cancer therapy and targeting CXCL12 (114). This small pro-inflammatory chemokine plays a significant role in tumor formation by binding to the specific receptor CXCR4. Using LBVS, Haider et al. identified three potential anti-cancer CXCL12 inhibitors (115). The above examples illustrate that LBVS holds extensive prospects for application. Its emergence provides researchers with an efficient, rapid, and cost-effective approach to identifying potential candidate compounds, thus advancing the development of computer-assisted research in this field.

#### 4.2.2 Structure-based virtual screening

SBVS approach relies on the three-dimensional structure of the target protein, often obtained through X-ray crystallography (116) or nuclear magnetic resonance (NMR) spectroscopy (117). SBVS is widely used to identify potential drug candidates and has proven to be an effective tool in the early-stage drug discovery process (118). It typically involves molecular docking, scoring, and filtering to prioritize compounds with high binding affinity and potential therapeutic activity against the target protein. Thus, SBVS significantly accelerates the drug discovery process by reducing the number of compounds that need to be experimentally tested (119). Here, we have presented numerous examples of drug screening based on SBVS, demonstrating its importance in enhancing drug screening efficiency and improving the accuracy of candidate drug selection. For instance, Mukherjee et al. discovered compound CID 88265020 with significant potential as a VGFR inhibitor among over 80 virtual screening compounds, which could be used for prospective future studies in ovarian cancer (120). Xie et al., based on the importance of FOXM1 in ovarian cancer treatment, selected XST-119 through SBVS and found that XST-119 exhibited apparent inhibitory activity in a xenograft mouse model, laying the foundation for further drug discovery (121). G9a, a lysine methyltransferase, was investigated by Bellver-Sanchis et al., who proposed a candidate G9a inhibitor, referred to as compound F., providing a lead for G9a inhibitor design and demonstrating their involvement in reducing Alzheimer’s disease (AD) (122). Guo et al. discovered novel Tropomyosin receptor kinases A (TrkA) allosteric inhibitors through SBVS and identified a promising hit (D5261, TrkA cell IC50 = 3.32 μM). Their results suggest that D5261 could be a starting point for developing TrkA allosteric inhibitors (123). Lin et al. reported a novel non-covalent Bruton tyrosine kinase (BTK) inhibitor that could potentially target malignant tumors, laying the foundation for developing effective BTK inhibitors for solid tumors (124). Luo et al. performed PD-L1 screening on 52,765 marine natural products and identified compound 51320 as a PD-L1 small molecule inhibitor through SBVS and other methods (125). Similarly, Ge et al. identified two compounds through SBVS that could serve as scaffolds for IDO1 inhibitors (126).

Furthermore, the SBVS process involves the interaction between molecules and proteins, making it indispensable for studying molecular mechanisms within living organisms. It contributes to uncovering the foundations of diseases and potential therapeutic pathways. In summary, with the continuous expansion of computer model training, SBVS is poised to become one of the critical tools in drug discovery.

### 4.3 Predicting the physicochemical properties and biological activities of compound

#### 4.3.1 Quantitative Structure-Activity Relationship

QSAR modeling is one of the most popular computer-aided tools in drug chemistry for drug discovery and lead compound optimization. It involves four main steps: selecting appropriate molecules, model construction, model validation, and model application (127). Since its introduction in the 1960s, QSAR modeling has been continuously refined and updated (128). One of the most representative 3D QSAR models is Comparative Molecular Field Analysis (CoMFA), considered the most classical and widely used. However, its development has been limited by certain drawbacks (129, 130). To address these limitations, Wang and colleagues created the Cloud 3D-QSAR server, which can facilitate the development of robust QSAR models in drug discovery (131). With the advancement of QSAR techniques, applying 4D, 5D, 6D, and 7D models is becoming more prominent, as these methods can provide additional information on small molecules beyond traditional 3D-QSAR (132).

#### 4.3.2 Predicting physical and chemical properties and biological activity

The physical and chemical properties of compounds determine their binding efficiency with targets, and predicting the physicochemical properties and biological activities of drugs can expedite the decision-making process.

Currently, numerous software programs are utilized for predicting the physicochemical properties and biological activities of drugs. JunctionTree VAE (Variational Autoencoder) is an open-source tool capable of predicting compound properties (133). Conv_qsar_fast is another open-source toolkit that uses CNN (Convolutional Neural Network) methods to predict molecular properties (134). Additionally, InnerOuterRNN utilizes internal and external recursive neural networks to predict physical, chemical, and biological properties (135). In conclusion, predicting the physicochemical properties and biological activities of drugs by AIDD can significantly enhance the efficiency and success rate of research and provide valuable tools and resources for scientists, accelerating the discovery and development of new medicines.

### 4.4 Prediction of compound toxicity

Toxicity testing is an essential but resource-consuming task for drug discovery (136), and utilizing AIDD in toxicity prediction can reduce the need for animal experiments (137, 138). To address this challenge, various tools have been developed. One of the most classic tools is Deep Tox, which employs deep learning methods to predict compound toxicity (139). Furthermore, eToxPred is a software capable of evaluating candidate compound toxicity based on machine learning methods (140). TargeTox can also predict drug safety related to toxicity (141). Therefore, compound toxicity prediction is crucial in drug development, contributing to improved research efficiency and risk reduction and providing robust support for drug safety assessment.

### 4.5 Drug repurposing

Drug repurposing, also known as drug repositioning (142), is categorized into early-stage and late-stage repurposing. The former involves the identification of potential lead compounds through large-scale screening of compound libraries (143); the latter is used to complete the preparation of lead compounds. For clinical testing and evaluation (144).

Drug repurposing has shown many successful examples: Aspirin was initially introduced by Bayer in 1899 as an analgesic and was first repositioned in the 1980s as an antiplatelet aggregation drug using low doses (145). With further research, scientists have revealed the therapeutic role of aspirin in the field of cancer, especially in colorectal cancer (146), where its anticancer effect is believed to result from the inhibition of COX-2, thereby blocking the anti-apoptotic effect of COX-2 in malignant cells and promoting their apoptosis (147). The second is Propranolol, originally a classic drug for treating angina and hypertension, which has recently been discovered to be effective in the treatment of osteoporosis and melanoma (148). As is well known, the chiral drug Sildenafil was once withdrawn from the market due to its teratogenic effects. However, a serendipitous discovery revealed that Sildenafil has a significant therapeutic impact on erythema nodosum leprosum (a type of autoimmune complication of leprosy) by inhibiting the synthesis of the pro-inflammatory cytokine tumor necrosis factor-alpha (TNF-α). Therefore, in 1998, it was repositioned by Celgene as an orphan drug for treating leprosy complications of leprosy (149). However, these cases are often based on serendipitous clinical discoveries and lack systematic patterns. Therefore, it is essential to utilize network-based approaches to discover the potential molecular mechanisms of drug actions (150).

Currently, there are several databases available for drug repurposing. For instance, the NCGC Pharmaceutical Collection (NPC) (http://tripod.nih.gov/npc/) is suitable for screening and research on numerous diseases, and it minimizes false negatives and false positives (151). DrugBank (www.drugbank.ca) is a network-based database that provides comprehensive molecular information about drugs, drug mechanisms, drug interactions, and their targets. It can offer crucial clues for drug repurposing (77). Moreover, Drugs@FDA and ClinicalTrial.gov provide the latest updates on drugs in clinical development, presenting new research directions and systematic applications in AIDD.

## 5 Big data improves the efficiency of adjuvant identification

The precision of AI-based drug screening is crucial to reduce the use of animal models. Big data technology and machine algorithms provide us with significant advantages. Optimizing AI models’ algorithms and parameters can improve predictive accuracy. Specifically, data preprocessing and standardization are essential (152). Standardizing and unifying data formats ensure comparability across different data sources. Additionally, addressing noise and missing values in the data can enhance its quality (153). On this foundation, model selection and extensive training are indispensable steps. Cross-validation and hyperparameter methods can improve model prediction accuracy (154, 155). Once we obtain raw data, converting it into features suitable for supervised learning, known as feature engineering, is necessary. This process includes feature creation, transformation, extraction, exploratory data analysis, and benchmarking, aimed at simplifying and accelerating data transformation while improving model accuracy (156). Finally, small-scale experimental validation of adjuvants generated by AI screening, followed by model adjustments based on the prediction results, can enhance screening precision. Currently, machine learning-based predictive models can leverage existing experimental and clinical data for training, thereby reducing the need for animal experiments (157).

## 6 Successful examples of different targets

**Figure 1:**
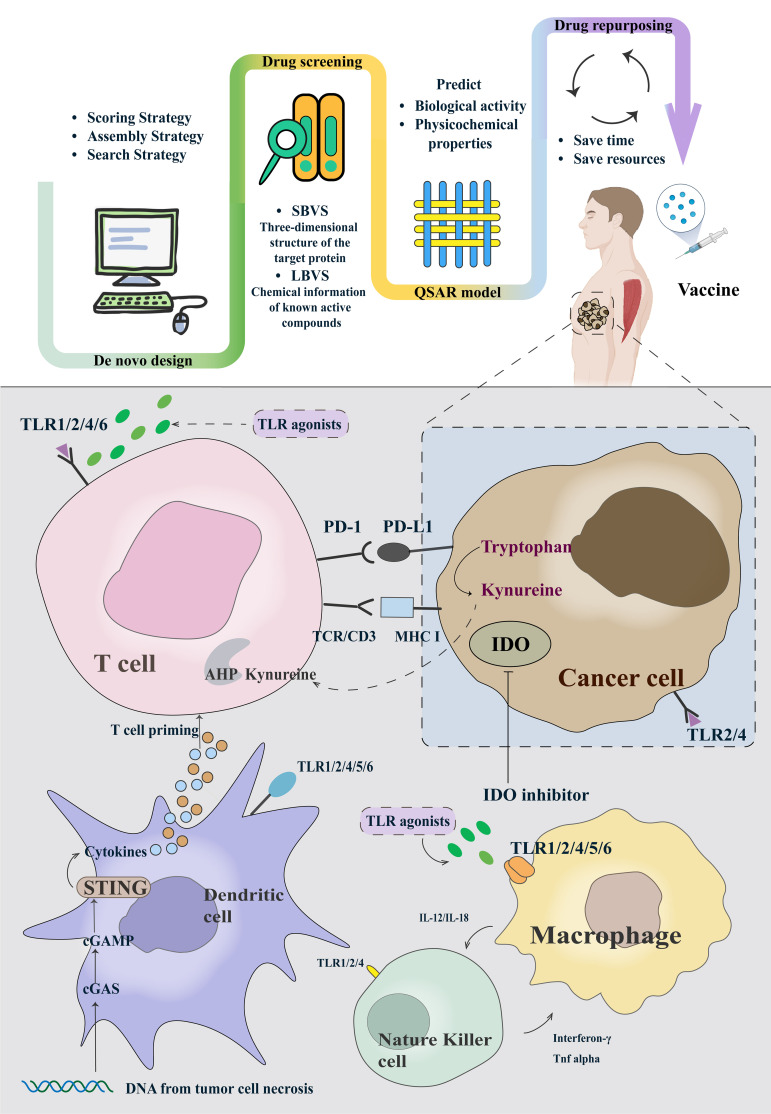
The framework of AIDD of adjuvants and typical examples of cancer vaccine adjuvants based on the clinical trials.

AI has aided researchers in streamlining the intricate process of identifying candidate compounds on various targets. Leveraging AI, novel adjuvants have been developed for several diverse functional targets (**Figure 1**). We will highlight the research progress of adjuvants in some of the most valuable target categories. The upper part of the figure, from left to right, describes how adjuvants are designed, screened, quantitatively structured, and eventually repurposed and added to vaccines. When a vaccine containing AI-screened adjuvants is injected into a patient, it triggers a series of immune responses, stimulating immune cells to produce and release cytokines or target and attack tumors.

### 6.1 Toll-like receptor family

The TLR family plays a crucial role in the innate immune system, typically serving as a class of pattern recognition receptors (PRRs) capable of recognizing specific molecular patterns associated with pathogens such as bacteria, viruses, and fungi (158). Specifically, when TLRs recognize one of these patterns, they trigger an immune response to protect the body from infection (159).

Due to these characteristics of TLRs, their agonists are widely used as adjuvants in cancer vaccines to enhance the immune response (14). We have summarized some of the relevant clinical advancements of TLRs in **Table 2**. Depending on the recognized molecular patterns, they can be categorized into four classes: Recognizing Bacterial Molecules: TLR1, TLR2, TLR4, TLR5, TLR6; Recognizing Double-Stranded RNA (dsRNA): TLR3; Recognizing Single-Stranded RNA (ssRNA) from Viruses: TLR7, TLR8; Recognizing Unmethylated CpG DNA Motifs from Viral DNA: TLR9. Some representative agents are examined below.

**Table 2:** TLR-based cancer vaccines adjuvant in clinical application.

Category	Biological	Status	Conditions	NCT code	Reasons for use as an adjuvant	AI tool
TLR3 agonist	Poly I: Poly C	Phase I/II	Melanoma, Ocular Melanoma, Uveal Melanoma	NCT04364230	Activates the TLR3 signaling pathway in the immune system and enhances anti-tumor immune responses	DeepChem
TLR3 agonist	Poly I: Poly C	Phase I/II	Melanoma, Metastatic Melanoma, Mucosal Melanoma	NCT02126579		Deep Neural Net QSAR
TLR3 agonist	Poly I: Poly C	Phase I/II	Melanoma	NCT01079741		DeepChem
TLR3 agonist	Poly I: Poly C	Phase I/II	Influenza, Human	NCT01591473		DeepChem
TLR4 Agonist	GLA-SE	Early Phase I	Skin Melanoma	NCT02320305	Stimulates the TLR4 signaling pathway and promotes anti-tumor immune responses	GeneWays
TLR4 agonist	MPL	Phase I/II	Melanoma, Ovarian Cancer, Lung Cancer	NCT01584115		
TLR7 agonist	R848 gel	Phase II	Melanoma	NCT00960752	Activates TLR7 signaling pathway and promotes antiviral immune response	PotentialNet
TLR7 agonist	GS-9620	Phase II	HIV/AIDS	NCT04364035		
TLR7 agonist	imiquimod	Phase I	Melanoma (Skin), Metastatic Cancer	NCT00453050		GeneWays
TLR7 agonist	R848	Phase I	Influenza, Vaccination in Seniors	NCT01737580		PotentialNet
TLR9 agonist	DUK-CPG-001	Phase II	Hodgkin Lymphoma, Non-Hodgkin Lymphoma	NCT02115126	Stimulate the TLR9 signaling pathway, enhances B-cell and dendritic cell activity	DeepTox
TLR9 agonist	CpG-7909	Phase I/II	Esophageal Cancer	NCT00669292		
TLR9 agonist	CpG	Phase I	Hepatitis B	NCT04843852		

#### 6.1.1 TLR 3 agonist

TLR 3 is a critical recognition receptor primarily identifying dsRNA, a hallmark of viral infection. It aids in combating viral infections mainly by activating the host’s innate immune response and is widely expressed in dendritic cells, macrophages, and some epithelial cells (160). TLR 3 adjuvants have been extensively studied for their ability to induce robust type I interferon responses, thereby enhancing vaccine efficacy. Numerous studies have reported the application of TLR3 agonists. For instance, some research suggests that polyinosinic polycytidylic acid (Poly (I:C)) as a vaccine adjuvant significantly enhances immune responses and shows promising anti-tumor effects in preclinical studies (161). RECEPTOR.AI offers a TLR 3 custom library predicted by Alphafold for on-demand applications, leveraging state-of-the-art virtual screening and parameter assessment technologies. They maintain a large virtual library with over 600 billion molecules to provide compounds with increased potency, selectivity, and safety. [RECEPTOR.AI Platform] (https://www.receptor.ai/platform).

#### 6.1.2 TLR 7/8 agonist

TLR7/8, expressed intracellularly, was first proposed in 2000 (162). They jointly recognize ssRNA, triggering the immune response to identify pathogens. TLR7 is primarily expressed in plasmacytoid dendritic cells (pDC), while TLR8 is mainly expressed in myeloid dendritic cells (163, 164). In recent years, TLR7/8 has gained popularity as an up-and-coming class of vaccine adjuvants due to their ability to directly activate antigen-presenting cells (APCs) and enhance cellular immunity.

Recent studies have utilized molecular dynamics simulations to investigate the selective mechanisms of structurally similar TLR7/8, which can be regarded as an advanced computational method (165). The integration of automation and data-driven design has further expanded the research parameters. By employing a combinatorial approach, TLR-7/8a binders that induce the desired T-cell immune responses and exhibit sufficient efficacy can be identified (166, 167). The Rapid Overlay of Chemical Structures software (vROCS version 3.1.1. OpenEye Scientific Software, Santa Fe, NM. [http://www.eyesopen.com]) is a tool used in computational chemistry and drug discovery. By comparing the shapes of millions of compounds, researchers can quickly narrow down potential candidates. Urban Švajger et al. employed the LVBS method to search for potential TLR7 ligands and discovered six new compounds, addressing the gap where only imidazoquinolines had been used as TLR7 modulators (168). To enhance the accuracy of flexible site binding, Duan et al. conducted enrichment evaluations of various virtual methods and proposed that a combination strategy can improve the effectiveness of virtual screening for TLR8 agonists (169).

However, a common drawback of TLR7/8 agonists is their reactogenicity. In recent years, many researchers have aimed to overcome this issue by encapsulating these small molecules in nanoparticles or covalently binding them to polymers. This approach can prevent harmful systemic reactions (170, 171). AI is also used to design nanomedicines that effectively deliver TLR7/8 agonists. Nanoparticle-based delivery systems can enhance the stability and targeting of these compounds, improving their efficacy and reducing side effects. This approach is particularly valuable in cancer therapy, where precise targeting of tumor cells is crucial. Research by Hyunjoon and colleagues reported that subcutaneous injection of biodegradable polymer, poly(d,l-lactide-co-glycolide) (PLGA), and nanoparticles containing TLR7/8 agonists resulted in a potent antigen-specific immune response. Their experiments validated these effects in various tumor models, demonstrating the potential of TLR7/8 as effective vaccine adjuvants for cancer immunotherapy (172).

Furthermore, studies have suggested that tethering small molecule TLR-7/8a to polymer scaffolds can enhance the immunogenicity of vaccines. The formed polymer-TLR-7/8a complexes can restrict the distribution of adjuvants and prolong their retention in lymph nodes, providing new insights for optimizing adjuvant design (172). All these findings underscore the potential therapeutic benefits of TLR7/8 as vaccine adjuvants.

#### 6.1.3 TLR9

The innate immune system in humans can be activated by bacterial DNA (173). This discrimination is attributed to mammalian DNA having a low frequency of CpG dinucleotides, most of which are methylated (174). Recent research has shown that unmethylated CpG sequences with two purines at the 5’ end and two pyrimidines at the 3’ end are necessary for immune activation (175, 176). TLR9 was discovered to specifically recognize the unmethylated CpG and initiated host immunity (177). Based on their immune-stimulatory functions, CpGs can be divided into CpG-A, CpG-B, and CpG-C. CpG-A mainly exists in double-stranded aggregates and comprises single CpG motifs with partial phosphorothioate (PS modification) and phosphodiester main chain bases. It is primarily responsible for activating the STING pathway, inducing the production of IFN-α and IFN-β by stimulating pDC, and can indirectly activate NK cells (178). CpG-B is composed of multiple CpG motifs entirely modified with PS. It can activate the TLR9 pathway to generate pro-inflammatory cytokines. Unlike class A, class B contains B cell activators and stimulates pDC maturation. CpG-C typically consists of fully PS-modified double-stranded palindromic motifs, exhibiting immunostimulatory effects similar to the first two classes; it can induce robust IFN-α production, pDC maturation, and effective B cell stimulation (179).

Many studies have shown that CpG Oligodeoxynucleotides (ODNs) can expedite vaccine responses. For example, the hepatitis B vaccine HEPLISAV-B, approved by the FDA in 2017, was the first vaccine to use CpG-ODN 1018 as an adjuvant. Compared to the traditional aluminum hydroxide adjuvant in Engerix-B^®^, HEPLISAV-B induced a faster and more sustained response and significantly improved immunogenicity (180). Furthermore, CpG-ODNs can induce Th1 responses, promote the generation of cytotoxic T lymphocytes (CTLs), and enhance the secretion of IFN-γ (181). Sayami et al. found that K3 (CpG-ODN) generated more antigen-specific antibodies than other TLR ligands and induced Th1 polarization. Their report on Transcutaneous immunization also indicated that K3 could promote B cell activation and differentiation, demonstrating the promising potential of K3 as a vaccine adjuvant (182).

In sum, with the continuous advancements in the PRR field, the strategic design of PRR-targeted adjuvants has emerged as a prominent area of research. This encompasses the combination and fine-tuning of methods. Such efforts hold promise for advancing the development of both preventive and therapeutic vaccines.

### 6.2 STING agonists

In recent years, Stimulator of interferon genes (STING) agonist stimulators have garnered significant interest as potential vaccine adjuvants and cancer therapeutics. The STING pathway is critical for immune responses against viruses and bacteria and contributes to antitumor immunity (183). It is an endoplasmic reticulum adapter that plays an essential role in the immune system (184). Cytoplasmic DNA activates the cyclic GMP-AMP synthase (cGAS)-STING pathway in this mechanism, triggering an immune response (185). DNA binding to cGAS activates its enzymatic activity, creating conditions for the formation of cyclic dinucleotide, 2’3’-cGAMP (186). This cyclic dinucleotide (CDN) can indirectly activate transcription factors in the STING pathway, including interferon regulatory factor 3 (IRF3) and nuclear factor-kappa B (NF-κB) (187).

Most STING agonists are CDN compounds, widely proposed as adjuvants in vaccine administration and cancer therapy (188). Leveraging the characteristics of STING agonists, Fu et al. combined CDNs with a cancer vaccine called STINGVAX, finding it effective in multiple mouse tumor models (189). CDNs as adjuvants for developing infectious disease vaccines were initially validated in pneumococcus and Staphylococcus aureus infections (190, 191). Since then, many teams have explored CDN applications in contagious diseases (192). Furthermore, nano adjuvants and nanomaterials have demonstrated significant advantages in vaccine design. For instance, CDN chemical modifications, such as fluorination and thiophosphorylation on the same side, have been found to activate STING with higher stability, cellular uptake, and immunostimulatory capacity for antitumor therapy (193). Additionally, combinations of nanoscale adjuvants have shown promise in enhancing innate immunity. For example, combining TLR7/8 and STING agonists as vaccine adjuvants encapsulated in polymer nanoparticles can further improve their effectiveness, demonstrating additive effects of adjuvant combinations (194).

However, clinical outcomes of STING agonists, whether used as monotherapy or combined with immune checkpoint inhibitors, have been disappointing (195–197). This indicates the presence of unknown mechanisms leading to adverse reactions. To go further, James J’s team revealed that reversing the methylation silencing of STING in mouse melanoma cell lines using clinically available DNA methylation inhibitors can enhance agonist-induced STING activation and type I IFN induction. This, in turn, can induce tumor regression in syngeneic mice through a CD8^+^ T cell-dependent immune response. These findings shed light on the mechanisms of how functional impairments in STING signaling within tumor cells lead to compromised responses to STING agonist therapy (198). Moreover, the clinical application of CDN-like compounds still faces a series of inescapable challenges. Due to their damaging charge property, strong water solubility, and high polar surface area, these compounds exhibit poor membrane permeability, potentially diminishing the effectiveness of vaccines when used as adjuvants (199). In **Table 3** we have also summarized the current clinical progress of STING as a vaccine adjuvant in clinical practice.

**Table 3:** STING-based cancer vaccines adjuvant in clinical application.

Category	Biological	Status	Conditions	NCT code	Reasons for use as an adjuvant	AI tool
CDNs	TAK-676	Phase I	Advanced or Metastatic Solid Tumors	NCT04420884	Anti-tumor immune response	DeepChem
	IMSA-101	Phase II	Solid Tumor	NCT04020185		PotentialNet
	BI1387446	Phase I	Solid Tumors	NCT04147234		DeepNeuralNetQSAR
	BMS-986301	Phase I	In participants with cancers that have failed to respond to T cell checkpoint-inhibiting antibodies	NCT03956680		DeepTox
	SB11285	Phase I	Advanced solid tumors	NCT04096638		PotentialNet
Non-CDNs Small Molecules	GSK3745417	Phase I	Advanced Solid Tumors	NCT03843359	Anti-tumor immune response	DeepChem
	SNX281	Phase I	Advanced Solid Tumors and Lymphoma	NCT04609579		GeneWays
	HG381	Phase I	Advanced Solid Tumor	NCT04998422		DeepTox
	KL340399	Phase I	Advanced Solid Tumors	NCT05549804		PotentialNet
	AZD6738	Phase II	Breast Neoplasm	NCT03740893	Anti-breast tumor immune response	DeepChem
Other	STAV	Phase I	Increase the body’s ability to fight aggressive relapsed or refractory leukemias.	NCT05321940	Respond to GeneWays zrelapsed or refractory leukemia	GeneWays
	SYNB1891	Phase I	Advanced/metastatic solid tumors and lymphoma	NCT04167137	Anti-tumor and anti-lymphoma immune responses	DeepTox

AI has made great progress in predicting and screening STING agonists as adjuvants. Ramanjulu et al. utilized high-throughput screening methods to discover ABZI (amidobenzimidazole). Intravenous injection of the STING agonist diABZI in immunocompetent mice with established syngeneic colon tumors resulted in complete tumor regression (200). Additionally, screening has shown that formulating STING agonists as nanoparticles can enhance drug stability and bioavailability (201).

### 6.3 Programmed Cell Death Protein 1/Programmed Death-Ligand 1 inhibitor

PD-1 and PD-L1 immune checkpoint molecules play a role in balancing immune responses in the immune system (202, 203). When excess PD-L1 is bound to PD-1 on T cells, it inhibits T cell activity, leading to a weakened immune response and immune escape (204, 205).

Typically, cancer vaccines stimulate the immune system to generate a specific response against the tumor (206). By introducing tumor-associated antigens, the vaccine can identify and target tumor cells. However, once a tumor has formed, the immune system is suppressed, limiting the therapeutic effectiveness of vaccines (207). In clinical practice, combining PD-1/PD-L1 inhibitors with tumor vaccines aims to enhance immunotherapy. PD-1/PD-L1 inhibitors as adjuvants relieve immune suppression, thereby strengthening the attack on tumors (208). There are examples in clinical trials that its antibodies and tumor vaccines have shown synergistic effects. For instance, Patrick A. et al. reported the first Phase Ib clinical trial (Clinicaltrials.gov: NCT02897765) using a neoantigen-based personalized vaccine called NEO-PV-01 in combination with PD-1 inhibitor Nivolumab to treat advanced melanoma non-small cell lung cancer, or bladder cancer (209). Compared to monoclonal antibodies, small molecules are cost-effective, have a shorter half-life, and can reduce sustained systemic side effects (210). Thus, they have become candidates for adjuvants. The design of small molecules targeting PD-1/PD-L1 typically involves disrupting protein-protein interactions (PPI) (211). Due to the lack of a prominent binding pocket, researchers have developed a series of biphenyl derivatives based on the mechanism of PD-1/PD-L1 interaction, including BMS-8, BMS-37, BMS-202, BMS-200, BMS-1001, BMS-1166 (212). PD-L1 dimerization is also a hot research area for small molecule PD-1/PD-L1 studies (213). For example, BMS’s small molecules tend to form stable dimers with PD-L1 monomers. BMS-1166 can specifically inhibit partial glycosylation of PD-L1, thereby blocking PD-L1 from moving to the Golgi apparatus from the endoplasmic reticulum, rendering it inactive (214). The first oral small molecule PD-L1 inhibitor that entered clinical trials is CA-170 (215). Its clinical trials have been conducted in lung cancer, head and neck cancer, and Hodgkin’s lymphoma (NCT02812875, clinicaltrials.gov) (CTRI/2017/12/011026, ctri.nic.in), which may mark a new era for small molecule vaccine adjuvants. In **Table 4**, we have summarized the clinical advancements of other PD-1/PD-L1 as small molecule adjuvants.

**Table 4:** PD-1/PD-L1-based cancer vaccines adjuvant in clinical application.

Category	Biological	Status	Conditions	Combination therapy	NCT code	Reasons for use as an adjuvant	AI tool
PD-1/PD-L1 inhibitor	CA-170	Phase I/II	advanced tumors and lymphomas	N/A	NCT02812875	Anti-tumor immune escape	DeepChem
	INCB-086550	Phase II	solid tumors	N/A	NCT04629339	Anti-tumor	PotentialNet
	MX-10181	Phase I	solid tumors	N/A	NCT04122339	Enhance ICBs therapy	DeepNeuralNetQSAR
	GS-4224	Phase I	solid tumors	N/A	NCT04049617	Tumor identification and clearance	DeepTox
	IMMH-010	Phase I	advanced solid tumors	N/A	NCT04343859	Anti-tumor	GeneWays

N/A, Not Available.

### 6.4 Indoleamine 2,3-dioxygenase inhibitor

IDO is an enzyme responsible for converting tryptophan into kynurenine. Within the immune system, the primary role of IDO is to suppress T-cell activity, helping to maintain immune balance and prevent excessive immune responses. In recent years, IDO inhibitors have drawn significant attention in tumor immunotherapy. As reported, it can delay tumor growth and enhance the effect of dendritic cell vaccines (216). They are often used with other immunotherapies, such as PD-1/PD-L1 inhibitors, to bolster the immune response. Yao et al. introduced an *in situ* multifunctional vaccine utilizing bacterial outer membrane vesicles (OMVs, 1-MT@OMV-Mal) designed to encapsulate IDO inhibitors internally. The *in situ* injection of 1-MT@OMV-Mal effectively overcomes the immune suppression induced by IDO on effector T cells infiltrating the tumor, resulting in significant inhibition of both primary and distant tumors (217). Su et al. used polymer-lipid hybrid nanovesicle (P/LNV)–based liposomes to deliver IDO inhibitors and TLR9, enhancing antigen immunogenicity and simultaneously blocking immune checkpoints. Mechanistically, this approach significantly increases the infiltration of CD8^+^ T cells in tumors and drains lymph nodes. Cationic liposomes delivered with tumor vaccines and IDO inhibitors provide a promising platform for cancer immunotherapy by provoking antitumor T-cell immunity and reversing the immunosuppressive tumor microenvironment (218). Troitskaya et al. demonstrated that IDO can enhance the efficacy of recombinant human milk peptide lactating (RL2) —treated cell vaccination. Their experimental results showed that additional IDO chemical inhibition exhibits better long-term antitumor responses than vaccination with RL2-treated cells alone (219).

Moreover, IDO as a cancer vaccine adjuvant also finds applications in the Human Papillomavirus (HPV) field. Recently, Pagni et al. developed a vaccine based on the genetic fusion of HSV-1 glycoprotein D (gD) with the HPV-16 E7 oncoprotein (gDE7 vaccine) for treating HPV-related tumors. They aimed to enhance the existing efficacy by incorporating an IDO inhibitor (206). *In vivo* experimental results showed that multitarget therapy improved the antitumor efficacy of the gDE7 protein vaccine, providing evidence for IDO as a therapeutic vaccine adjuvant for HPV tumors (220). Their earlier studies also indicated the significant role of IDO in HPV vaccines. They added two immune metabolic adjuvants, an IDO1 inhibitor, to the gDE7 vaccine and found that they enhanced the *in vivo* antitumor effect. In simple terms, combining IDO inhibitors with immunotherapy and reducing the negative impact of IDO1 expression on vaccine-induced protective immunity significantly increased the anticancer effect (221). Their experiments demonstrated that IDO1-targeted therapy could improve antitumor treatment by reprogramming inflammatory cells. These series of studies revealed a novel and promising approach to control HPV-related tumors and potentially other cancer types, providing substantial evidence to support further research.

Finally, a few IDO inhibitor has been tested in clinical. For example, Indoximod has been widely used in clinical settings. A phase I/II clinical trial (NCT01042535) combining indoximod with adenovirus p53-Dendritic Cells (DC) vaccine in the treatment of invasive breast cancer showed a maximum tolerated dose (MTD) of 1600 mg BID, and the two drugs demonstrated significant synergistic effects. This proves that IDO inhibitors can effectively enhance the efficacy of vaccines (222). Therefore, the strategy of using indoximod as an adjuvant for tumor vaccines to enhance anti-tumor treatment is worthy of further exploration in subsequent clinical trials. In sum, we summarized the clinical trials of potential IDO inhibitors as adjuvants for tumor vaccines currently underway and presented them in **Table 5**.

**Table 5:** IDO-based cancer vaccines adjuvant in clinical application.

Category	Biological	Status	Conditions	Combination therapy	NCT code	Reasons for use as an adjuvant	AI tool
IDO inhibitor	Epacadostat(INCB024360)	Phase III	Cisplatin-ineligible Urothelial Carcinoma	Pembrolizumab、 Epacadostat and Placebo	NCT03361865	Anti-tumor immune escape	DeepChem
IDO inhibitor	GDC-0919	Phase I	Solid tumor	N/A	NCT02048709	Anti-tumor	PotentialNet
IDO inhibitor	1-methyl-D-tryptophan	Phase I	Solid tumor	N/A	NCT00739609	Inhibits IDO and increases T cell activity	DeepNeuralNetQSAR
IDO inhibitor	NLG802	Phase I	Solid tumor	N/A	NCT03164603	Anti-tumor	DeepTox
IDO inhibitor	Docetaxel	Phase II	Metastatic Breast Cancer	Indoximod in Combination With a Taxane Chemotherapy	NCT01792050	Anti-tumor	PotentialNet
IDO inhibitor	KHK245	Phase I	Locally Advanced or Metastatic Urothelial Carcinoma	KHK2455 in combination with avelumab	NCT03915405	Enhanced immunotherapy	DeepChem
IDO inhibitor	Indoximod	Phase I/II	Temozolomide-Refractory Primary Malignant Brain Tumors	Indoximod 、 Temozolomide and Bevacizumab	NCT02052648	Synergistic effect of chemotherapeutic agents	GeneWays
IDO inhibitor	Indoximod	Phase I/II	Metastatic Melanoma	Indoximod、 Ipilimumab、 Nivolumab and Pembrolizumab	NCT02073123	Enhance ICBs therapy	DeepTox
IDO inhibitor	Indoximod	Phase I/II	Metastatic Adenocarcinoma of the Pancreas	Indoximod in Combination With Gemcitabine and Nab-Paclitaxel	NCT02077881	Anti-tumor	DeepChem
IDO inhibitor	Indoximod	Phase I	Progressive Primary Brain Tumors	Indoximod and Temozolomide	NCT02502708	Improving the efficacy of chemotherapy drugs	PotentialNet
IDO inhibitor	Epacadostat(INCB024360)	Phase II	Advanced Melanoma	Indoleamine 2,3, Dioxygenase-1 (IDO1) Inhibitor (INCB024360) Plus a Multi-peptide Melanoma Vaccine (MELITAC 12.1)	NCT01961115	polypeptide vaccine	DeepNeuralNetQSAR
IDO inhibitor	Epacadostat	Phase III	Metastatic Disease	Pembrolizumab、 Epacadostat	NCT03374488	Enhance ICBs therapy	DeepTox
IDO inhibitor	Epacadostat	Phase II	Non-Small Cell Lung Cancer	Pembrolizumab (MK-3475) Plus Epacadostat (INCB024360)	NCT03322540		PotentialNet
IDO inhibitor	Epacadostat	Phase II	Non-Small Cell Lung Cancer	Pembrolizumab、 Epacadostat、 Platinum-based chemotherapy	NCT03322566		DeepChem
IDO inhibitor	Epacadostat	Phase I/II	Solid Tumors	Epacadostat、 Pembrolizumab and Oxaliplatin	NCT03085914		GeneWays

N/A, Not Available.

## 7 Conclusion

The development of new drugs is a lengthy and expensive process. The average cost of research and development is around $1.3 billion per drug (223). The average duration for developing oncology drugs is 13.1 years, with only 13.8% of all drug development projects ultimately gaining approval (224, 225). These challenges are often attributed to inappropriate patient selection, outdated equipment, and technological limitations. With the application of AI in drug development, it is possible to reduce these figures (226). For example, AI can use patient genetic exposure profiles to screen for potential drug target populations (226). Predictive machine learning and alternative deductive methods also contribute to forecasting lead compounds, thereby reducing development costs (226).

**Figure 2:**
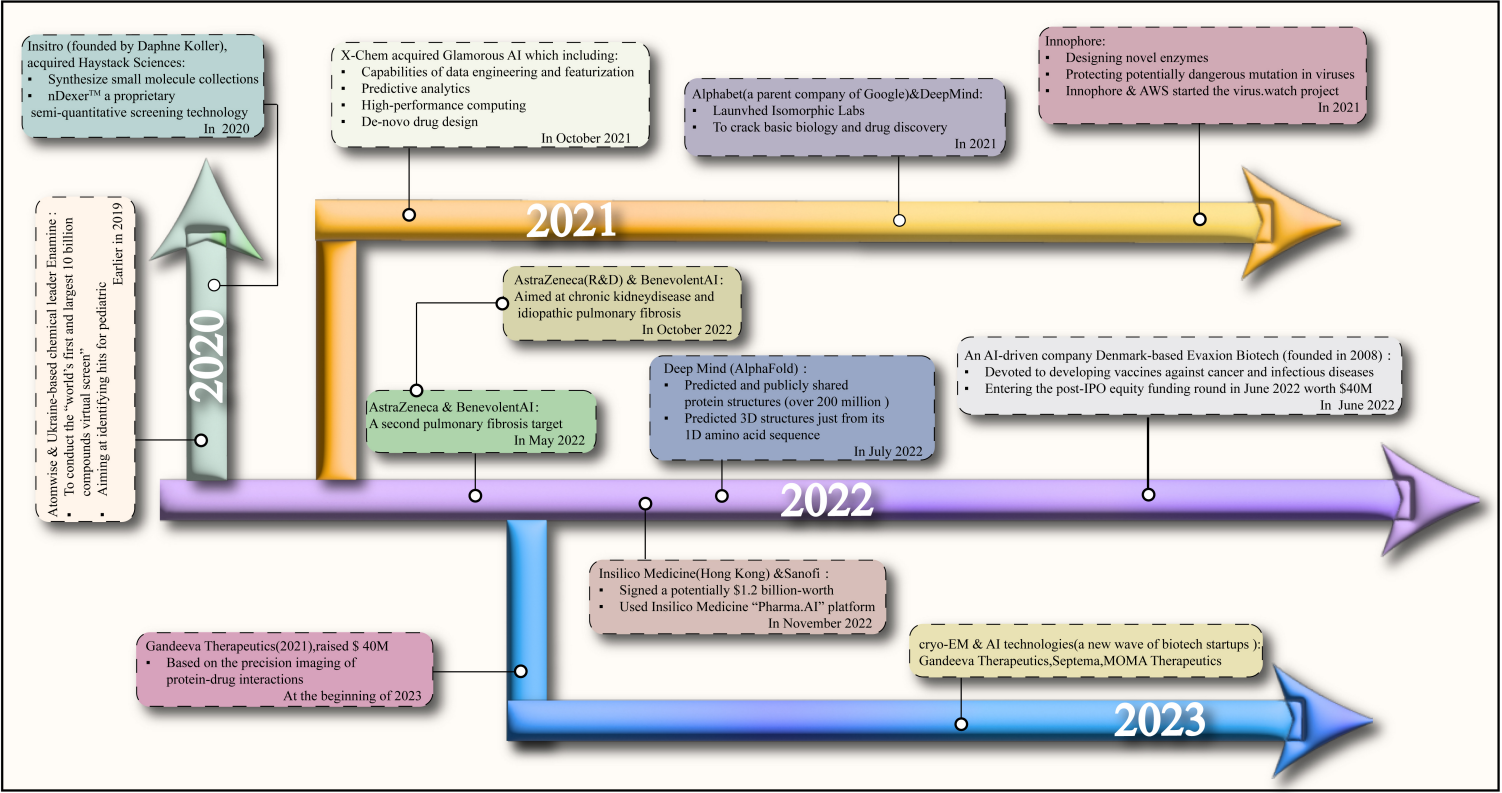
The streamlining of the process of bringing drugs into clinical trials and the collaboration between artificial intelligence companies and pharmaceutical companies based on the years.

To reduce the costs associated with drug development, there is an increasingly close collaboration between pharmaceutical companies and the field of AI, especially in the realm of target discovery. We have compiled a summary of projects launched in partnership between select AI companies and pharmaceutical firms from 2020 to the present. These initiatives aim to develop and predict viable drugs, ultimately streamlining the process of bringing drugs into clinical trials (**Figure 2**). Current AI systems are mainly based on the deep learning method, the most popular machine learning method after the success of deep neural network (DNN) (227). Deep learning has been widely explored in various tasks (228–230), including drug design and molecular dynamics (MD) development (231). The MD development plays a crucial role in the small molecule adjuvant effect since it can explain how molecules interact at the atomic level during drug discovery (232). However, the MD process is known to be time-consuming and labor-intensive. The AI-based system can solve these issues and thus accelerate the MD process. For example, Drew Bennett et al. proposed an MD simulation that calculates the free energy of transferring 15,000 small molecules from water to cyclohexane (233). The research findings show that AI technology can speed up the MD simulations and enhance improvisation, however, at the cost of substantial model training, which is indispensable for deep learning-based AI systems.

The success of AI in developing adjuvants for cancer vaccines relies heavily on the availability of extensive data and well-trained models (2). Accessing various databases has introduced additional costs, and screening reliable and high-quality data is essential to ensure prediction accuracy. AI faces significant challenges in developing adjuvants for cancer vaccines and other drugs. These challenges include reducing the 2-3 year gap between prediction and drug development, a shortage of skilled personnel for AI-driven drug screening, ensuring the credibility of generated predictions, dealing with the “black box” phenomenon, and establishing reasonable regulations (234). While AI has been widely used in drug discovery and translational research over the past two decades, its progress in clinical operations and data analysis has been relatively slow.

While many challenges are associated with using AI in developing adjuvants for cancer vaccines, we anticipate that AI will become increasingly prevalent in this field, helping mitigate the risks associated with drug development (235, 236). With the development of personalized medicine, AI can also assist in selecting the most suitable adjuvants for patients to achieve personalized immunotherapy plans. This will contribute to the advancement of precision medicine. Overall, AI has accelerated the research process in developing tumor vaccine adjuvants, improving the efficiency and effectiveness of adjuvant design. It can bring more innovation and breakthroughs to the future of global vaccine research and immunotherapy.
